# Differential expression, localization and activity of MARCKS between mantle cell lymphoma and chronic lymphocytic leukemia

**DOI:** 10.1038/bcj.2016.80

**Published:** 2016-09-23

**Authors:** J Vargova, K Vargova, N Dusilkova, V Kulvait, V Pospisil, J Zavadil, M Trneny, P Klener, T Stopka

**Affiliations:** 1 Biocev, First Faculty of Medicine, Charles University, Vestec, Czech Republic; 2 Group of Molecular Mechanisms and Biomarkers, International Agency for Research on Cancer, Lyon, France; 3 Department of Hematology, General Faculty Hospital, Prague, Czech Republic

Mantle cell lymphoma (MCL) is an aggressive and often-relapsing disease characterized by the clonal proliferation of CD5^+^ antigen-naive pre-germinal center B cells that form solid tumors and also enter the peripheral blood through a process called leukemization. MCL cells overexpress cyclin D1 owing to a *t*(11;14) chromosomal DNA translocation, although there also exist few MCL cases lacking these biomarkers.^1^ MCL co-express CD19, CD20 and CD5 antigens with chronic lymphocytic leukemia (CLL), which has many more indolent clinical outcomes, and is typically marked with leukemization. Some antigens (CD23 and CD200) are expressed in CLL, however, not in all cases.^2^ In addition, few CLL cases also contained the *t*(11;14) translocation.^3^ Overlapping and disease-specific features are not always reliable to distinguish between MCL and CLL, and this requires identification of additional biomarkers. MCL from CLL were not yet explored by comprehensive global approaches, despite such understanding possibly being very neat for deciphering pathogenesis and tailoring therapies of these clinically distinct diseases. Undertaking such studies is supported by recently identified CLL-upregulated RNAs for LEF1^(ref. 4)^ or microRNA miR-155;^5^ or SOX11, being overexpressed in MCL, but not in CLL.^2^

We herein utilized a global approach to identify specific expression differences in samples from the MCL and CLL patient test groups (*N*^MCL^=10, *N*^CLL^=11), and normal control subjects (*N*^NBC^=8; Supplementary Tables S1 and S2). The hierarchical clustering analysis used all significantly deregulated probes from the Affymetrix Human Genome HG-U133 Plus 2.0 Array, hybridized with magnetically purified CD19^+^ complementary RNA (Supplementary Methods), and grouped all MCL samples within a dendrogram that was clearly separated from the second branch of CLL samples. Although the third branch contained only normal B cells (NBCs) (Supplementary Figure S1), except one CLL patient sample from a partial remission (CLL01, containing a mixture of normal and tumor cells). The transcriptomic signatures from MCL patients were separated from controls (and CLL) also using the principal component analysis (Supplementary Figure S2). A similar strategy of utilizing DNA arrays for biomarker discovery proved to be very efficient and reliable on other types of lymphomas.^6^

The comparative analyses (Supplementary Methods) identified a set of 892 differentially expressed genes between MCL and NBC (260 upregulated and 632 downregulated). The MCL-specific biological processes included the immune system, cell activation, and response to stimulus and stress (Supplementary Table S3). The MCL-specific messenger RNAs (mRNAs) including those on the top and previously connected with MCL pathogenesis, such as Cyclin D1, SOX11 or WNT3, are listed in the Supplementary Tables S4 and S5. ‘MicroRNAs in cancer' represent one of the top MCL-specific pathways (Supplementary Table S6) supporting the role of deregulated expression of microRNAs and their targets in MCL. Similarly to MCL, we also investigated the transcriptomic signature of CLL patients. The comparative analyses (Supplementary Methods) identified a set of 774 differentially expressed genes between CLL and NBC (337 upregulated and 437 downregulated). The CLL-specific biological processes include the regulation of response to stimulus, immune system processes, and actin filament bundle assembly and organization (Supplementary Table S7). Among the most deregulated CLL-specific pathways were again the ‘MicroRNAs in cancer' (Supplementary Table S8), underlining the role of deregulated expression of microRNA targets also in CLL.

To search for MCL-/CLL-specific biomarkers, we noted 222 mRNAs, from which 216 were changed in the same direction, whereas 6 mRNAs were deregulated in the opposite direction between MCL and CLL, which implicates their common and unique pathogenic roles (Figure 1a). The set of six disease-specific mRNAs contained previously reported biomarkers: CD200,^2^ LEF1,^4^ CRIM1,^7^ Titin,^8^ an unknown RNA, and finally the myristoylated alanine-rich C-kinase substrate (MARCKS) that has not yet been studied in MCL. MARCKS encodes for an 87 kDa protein containing three functional domains: membrane-associated myristoylated N-terminal domain, MH2 domain and also a phosphorylation domain that is recognized by protein kinase C (PKC), calmodulin, actin or phosphatidylinositol bisphosphate PIP2.^9, 10^

The gene expression data indicated that MARCKS mRNA is threefold upregulated in MCL vs NBC and fourfold downregulated in CLL vs NBC (Supplementary Figure S3a). Next, we utilized the validation patient groups (*N*^MCL^=6, *N*^CLL^=8; Supplementary Tables S9 and S10) to study MARCKS expression at protein levels by flow cytometry and confirmed that MCL cells expressed significantly higher level of MARCKS compared with CLL samples (Supplementary Figure S3b).

As MARCKS was previously showed either bound to the cell membrane or reside in the cytosol, or alternatively become transmitted to nuclei via PIP2,^11^ we investigated subcellular localization of MARCKS using immunofluorescence (IF). Indeed, the overall signal between MCL and CLL was markedly higher in MCL. In addition, the MCL-MARCKS was localized mostly in the cytoplasm, whereas the CLL-MARCKS and NBC-MARCKS were localized in both cytoplasm and nucleus (Figure 1b). The cytoplasmic signal in MCL was significantly higher than in CLL, whereas the opposite was observed for the nuclear IF signal (Figure 1c). The ratio between cytoplasmic and nuclear signals was 2.5 for MCL and 0.8 for CLL (Figure 1c; *P*<0.0001). This pattern was observed in all patients from the validation group except the two MCL patients (MCL15 and MCL16) that contained 87% of the non-clonal population within the peripheral blood (Supplementary Figure S4).

The active forms of MARCKS are phosphorylated on serine residues by PKC^12^ mediating the oncogenic effects.^13^ This contention is supported by another study demonstrating that phosphorylation of MARCKS mediates cancer invasiveness^14^ in a PKC-dependent manner.^15^ We therefore investigated the abundance of two previously tested residues, phosphoMARCKS (pMARCKS)^Ser162^ and pMARCKS^Ser159/163^, in MCL and CLL. Signal distribution for pMARCKS^Ser162^ was strictly cytoplasmic and its abundance was very similar between MCL and CLL samples (Figure 2a). The signal distribution of pMARCKS^Ser159/163^ in MCL was again cytoplasmic but also partly nuclear (Figure 2a). In contrast to pMARCKS^Ser162^, the pMARCKS^Ser159/163^ cytoplasmic signal in MCL was markedly higher compared with CLL (in which it was rather nuclear) (Figure 2a) implicating that the residue Ser159/163 is a hyperphosphorylated form in the MCL cytoplasm, and its level and distribution markedly differ from CLL or NBC (Figure 2b).

We next searched for regulatory mechanisms upstream of the MARCKS expression in MCL vs CLL. We noted that microRNA pathways were deregulated in MCL (Supplementary Table S6) and CLL (Supplementary Table S8), and this also involved MARCKS (Supplementary Table S11). We hypothesized that MARCKS may be a target of microRNAs regulating gene expression by binding to the 3′-untranslated region of the target mRNAs to cause transcript degradation or to interfere with the translation initiation. As expected, MARCKS is a predicted target of several microRNAs using the DIANA Tools (http://diana.imis.athena-innovation.gr/DianaTools/index.php?r=site/page&view=software), and among them also of miR-155 (that is differentially expressed between MCL and CLL) with three 12 nt homologies based on miRanda predictions.^5^ As expected, a trend to a negative correlation between miR-155 and MARCKS was observed in CLL (*r*=−0.418), but not in MCL (*r*=0.046; Supplementary Figure S5), suggesting that miR-155 inhibits MARCKS expression in CLL. To investigate this possibility, we utilized a CLL cell line MEC-1, and using the CRISPR/Cas9 technology, we prepared individual cell clones (Supplementary Methods) with mutated miR-155 recognizing the MARCKS mRNA. From the miR-155 sequence mutants, we selected the indels that disrupted 17 out of 23 nt of the mature sequence (Supplementary Figure S6). We validated the heterozygous monoallelic mutation (MM) as well as biallelic mutations (BMs) of the miR-155 sequence using Sanger sequencing. Next, we utilized IF to determine the MARCKS level and localization in these clones. As expected, the BM-miR-155 MEC-1 cells expressed markedly higher level of MARCKS compared with MM-miR-155 or non-modified MEC-1 cells as determined by flow cytometry (Supplementary Figure S7). Differences in MARCKS expression prompted us to determine its subcellular localization in the BM-miR-155, MM-miR-155 and MEC-1 cells. We observed that upon loss of the miR-155 mature sequence, the level of cytoplasmic MARCKS significantly increased in the BM-miR-155 mutants (Figure 2c). Interestingly, the level of nuclear MARCKS also increased in the BM-miR-155 mutant, so the ratio between nuclear and cytoplasmic signals remained the same (Figure 2c). To summarize this part, the mutagenesis experiments with the miR-155 in CLL cells allowed us to conclude that the MARCKS level is at least in part controlled by the oncogenic miR-155.

In conclusion, our work identified a set of six differentially expressed biomarkers for MCL and CLL, and among them, MARCKS to be differentially expressed, localized and phosphorylated between MCL and CLL, this being partly controlled by oncogenic microRNA miR-155. MARCKS may have an important role in the MCL pathogenesis and can serve as a useful MCL biomarker.

## Figures and Tables

**Figure 1:**
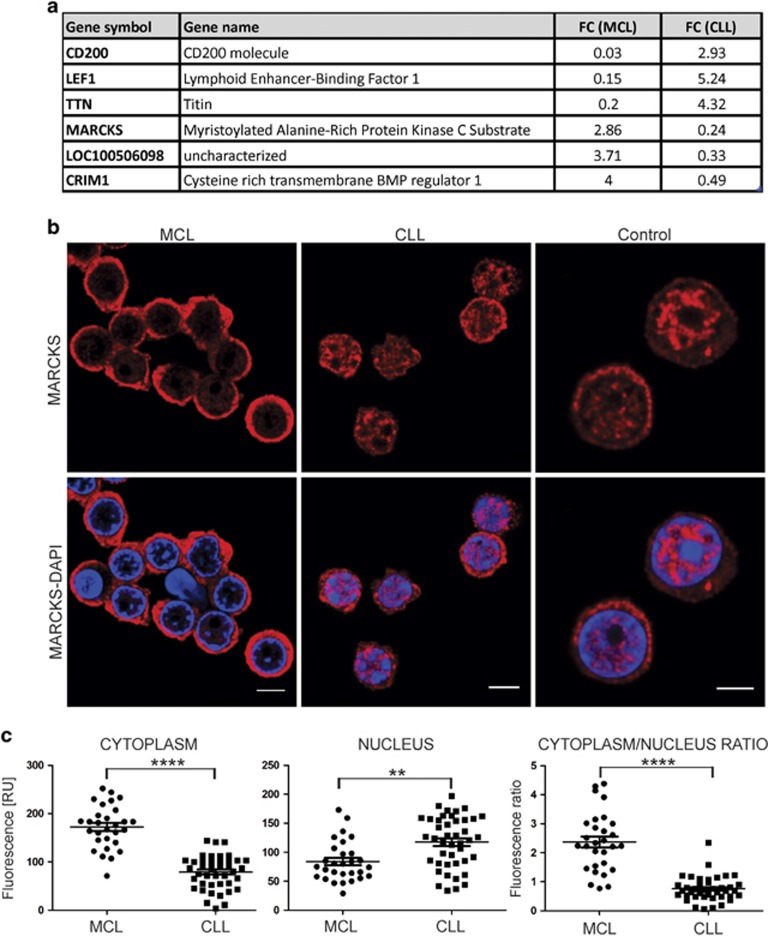
MARCKS expression and localization in MCL and CLL. (**a**) List of six genes significantly deregulated in both diagnoses in opposite directions. Fold change (FC) to normal controls. (**b**) Localization of MARCKS in peripheral blood mononuclear cells (PBMC) of MCL and CLL patients from the validation group and healthy controls. Cells were fixed and fluorescently labeled for MARCKS. DAPI was used for nuclear staining. Scale bars represent 5 μm. (**c**) Fluorescence intensity of the anti-MARCKS antibody in the cytoplasm, nucleus and its ratio determined by IF in PBMC of MCL and CLL patients from the validation group. Results of Student's *t*-test are displayed. **P⩽0.01, ****P⩽0.0001.

**Figure 2:**
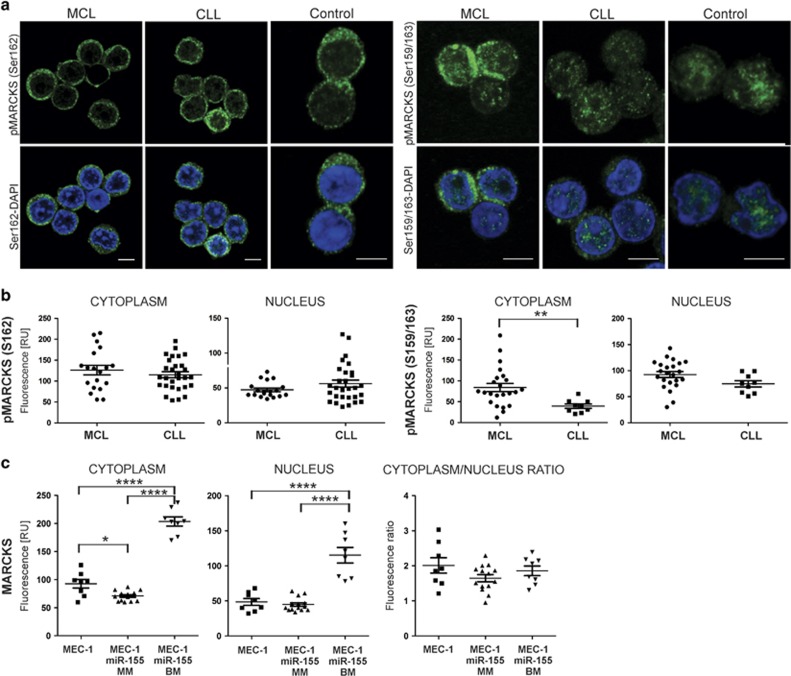
(**a**) Localization of phosphoMARCKS (pMARCKS) phosphorylated at Ser162 and Ser159/163 in PBMC of MCL and CLL patients from the validation group and healthy controls. Cells were fixed and fluorescently labeled for pMARCKS at Ser162 or Ser159/163. DAPI was used for nuclear staining. Scale bars represent 5 μm. (**b**) Fluorescence intensity of the anti-pMARCKS (Ser162) antibody and anti-pMARCKS (Ser159/163) antibody in the cytoplasm and nucleus determined by IF in PBMC of MCL and CLL patients from the validation group. (**c**) Fluorescence intensity of the anti-MARCKS antibody in the cytoplasm and nucleus (and its ratio) in MEC-1 cell line, and miR-155 clones determined by IF. Each dot represents one cell. Results of Student's *t*-test and Tukey's honest significant difference statistical test are displayed. *P⩽0.05, **P⩽0.01, ****P⩽0.0001. MM, monoallelic mutation; BM, biallelic mutation.
