# Supplementary material for: Differential expression, localization and activity of MARCKS between mantle cell lymphoma and chronic lymphocytic leukemia
Source: Blood Cancer J. 2016 Sep 23;6(9):e475–. doi: 10.1038/bcj.2016.80 (PMC5056972; doi:10.1038/bcj.2016.80)
Supplement: Supplementary Information [file bcj201680x1.pdf]

SUPPLEMENTAL METHODS

Patients

Test group of patients included peripheral blood cells (PBMC) collected in years 2009–2012 from patients with MCL (N = 10, median age 66 years), CLL (N = 11, median age 72 years), and healthy controls (N = 8, median age 33 years), following written informed consent based on Helsinki declaration and approved by Institutional Ethics Committee. MCL samples were collected at the time of diagnosis (N=8) and from previously treated patients (N=3). As a control, one sample represented a patient in partial remission. The median percentage of clonal B-lymphocytes was 60% (range 17-94). PBMC from CLL were sampled at diagnosis (N=2), relapse (N=1), or progression (N=7). Again, one sample represented a partial remission. In treated samples, the chemotherapy was administered at least 6 months before the time of sampling. Patient data are summarized in the Supplemental Tables 1 & 2.

Validation group of patients included PBMC collected in years 2015-2016 from patients with MCL (N=6, median age 63 years) and CLL (N=8, median age 65 years). Samples represented untreated patients at the time of diagnosis, shortly prior therapy application. The median percentage of clonal B-lymphocytes in MCL group was 78% (range 13-97%). Patient data are summarized in the Supplemental Tables 3 & 4.

PBMC were isolated by fractionated Ficoll-Paque gradient (GE Healthcare, Pollards Wood, UK). Subsequently, the CD19+ fraction of B-cells was separated from patients' PMBC as well as from the normal healthy donor PB samples by magnetic beads (MACS, Miltenyi Biotec, Cologne, Germany). The cell purity and viability always exceeded 90 % as determined by flow cytometry (FACSCanto II, BD Biosciences, San Jose, CA) and trypan blue staining, respectively.

Supplemental methods

DNA microarrays

Total RNA was isolated using RNeasy kit (Qiagene); its integrity was determined by 2100 Bioanalyzer (Agilent Technologies, Santa Clara, CA). Samples with RIN ≥ 8,5 were included in the study. 1µg of total RNA was used to prepare biotin-labeled cRNA according to Affymetrix 3´-IVT Express Kit protocol. cRNA probes were used for subsequent hybridization on the gene expression chips (Affymetrix Human Genome HG-U133 Plus 2.0 Array containing nearly 50.000 probes), followed by fluorescent staining, fluidics processing, and scanning according to the Affymetrix recommendations. Microarrays data were summarized using RMA (Robust Multi-Array Average) algorithm. For differential gene expression analysis, an approach from the Limma package was used. Probes were accepted as differentially regulated on FDR adjusted *P*-value≤0.01 and $|\log_2 FC| \geq 1$. The significantly impacted pathways and biological processes were analyzed using Advaita Bio's iPathwayGuide (http://www.advaitabio.com/ipathwayguide). Principal component analysis was performed using the package Affycoretools and graphical output was created using plotPCA function. MultiExperiment Viewer MeV4.0 software was used for hierarchical cluster analysis (10).

Immunofluorescence

PBMCs from validation group and MEC-1 miR-155 mutants were fixed (2% PFA, 20 minutes), cytospined on slides, permeabilized (90% methanol, 30 minutes on ice) and blocked with 3% non-fat milk/PBS for 10 minutes at room temperature. For immunostaining the primary antibody anti-MARCKS (ab55451, Abcam, Cambridge, UK, dilution 1:50, at 4°C overnight) and anti-MARCKS (phospho-S162, phospho-S159/S163) (ab194804 and sc-12971-R, dilution 1:100, at 4°C overnight) were used. The primary antibody was detected by secondary antibodies coupled either with Alexa Fluor 488 (711-546-152,

Jackson ImmunoResearch Laboratories, West Grove, PA, dilution 1:200, at room temperature for 1 hour) or Alexa Fluor 594 (A-11020, Invitrogen, dilution 1:200, at room temperature for 1 hour). Following each of the antibody incubation, cells were repeatedly washed in 0.1% Tween 20-PBS. The fixed and immunostained samples were mounted with Vectashield Mounting Medium (Burlingame, CA) containing 4',6-diamidino-2-phenylindole (DAPI). Images were acquired using confocal laser scanning microscope Leica TCS SP2 with AOBS system (oil immersion 63x objective N.A. 1.4). Digital processing of images was performed using ImageJ 1,46r software (11). Y-axis in intensity profile graphs represents fluorescence of respective fluorophor and X axis represents distance.

Flow cytometry

Fixed (2% PFA, 20 minutes) and permeabilized (90% methanol, 30 minutes on ice) PBMC from validation group and MEC-1 miR-155 mutants were blocked (0,5% BSA-PBS) and immunostained with primary antibody to MARCKS (ab55451, Abcam, 5ul/10^6 cells, at room temperature for 1 hour) and then with secondary antibody Alexa Fluor 594 (A-11020, Invitrogen, 1ul/reaction, at room temperature for 30 minutes). Mean fluorescence intensity (MFI) was determined by BD FACSAria IIu (BD Biosciences).

CRISPR/Cas9

Expression vector U6gRNA-Cas9-2A-GFP, containing guide RNA for mature miR-155, sequence for Cas9 protein and selection marker GFP, was purchased from Sigma/Merck (Darmstadt, Germany). MEC-1 cells (CLL cell line; kind gift from Dr. M. Mráz, Brno, CZ) were transfected by nucleofector Amaxa (Lonza; program U-015; 2x10^6 cells/reaction) together with 1µg of expression vector U6gRNA-Cas9-2A-GFP,

according to the manufacturer's recommendations. After 24 hours post-transfection, the viable (PI negative) and GFP positive cells were sorted with FACSAria IIu as single cells into the 96 well plate and cultured in IMDM medium supplemented with 10% FBS, 1% Penicillin/Streptomycin mix at 37°C with 5% CO_2 for 2 weeks. To validate the genome editing, endpoint PCR followed by the Sanger sequencing of the PCR product were performed. In parallel, the RNAs from CRISPR/Cas9/miR-155 mutants were extracted to confirm levels of miR-155.

Statistical analysis

The software package R Version 3.2.1 (www.r-project.org), NET programming framework (C#), Microsoft MS SQL database, GraphPad Prism 5 and Microsoft Excel were used. A one-way ANOVA followed by Tukey's HSD was performed to compare multiple groups. A Student's *t* test was used for two groups comparison. A simple regression model was used to study the relationship between miR-155 and MARCKS expression. All statistical tests were two-sided, and P values < 0.05 were considered as statistically significant.
