# Supplementary material for: Differential expression, localization and activity of MARCKS between mantle cell lymphoma and chronic lymphocytic leukemia
Source: Blood Cancer J. 2016 Sep 23;6(9):e475–. doi: 10.1038/bcj.2016.80 (PMC5056972; doi:10.1038/bcj.2016.80)
Supplement: Supplementary Figures [file bcj201680x2.pdf]

SUPPLEMENTAL FIGURES

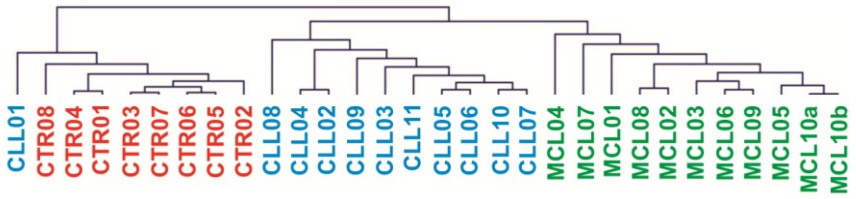

Supplemental Figure 1: Dendrogram of hierarchical cluster analysis based on the gene expression profiles of MCL, CLL and healthy controls samples generated by Multiple Array Viewer.

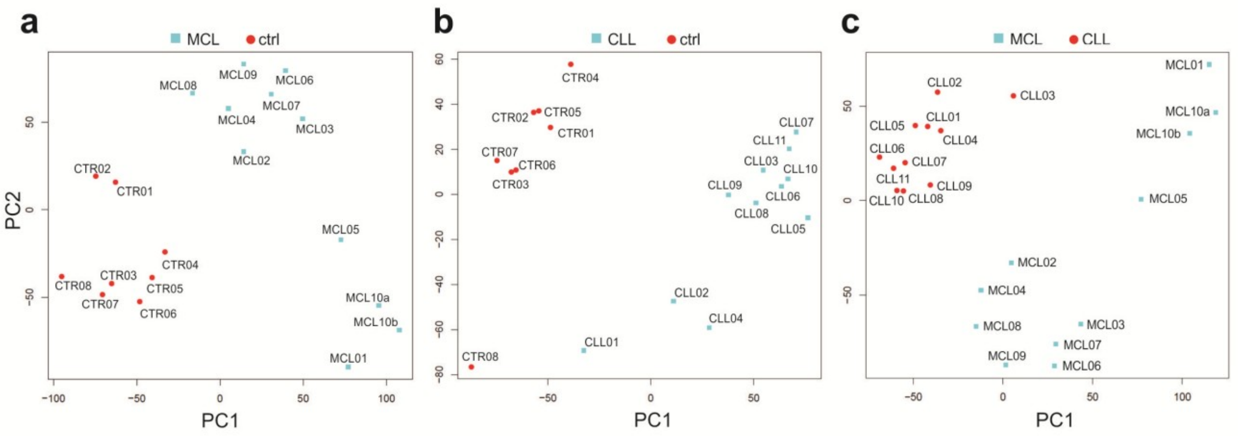

d

Code	Gender	Age	Status	Th prior the sampling
**MCL01**	F	64	PR	yes
**MCL02**	F	84	new Dg	no
**MCL03**	M	66	new Dg	no
**MCL04**	M	60	new Dg	no
**MCL05**	M	57	new Dg	no
**MCL06**	M	57	new Dg	no
**MCL07**	M	76	new Dg	no
**MCL08**	M	68	new Dg	no
**MCL09**	M	41	new Dg	no
**MCL10a**	M	71	relapse	yes
**MCL10b**	M	71	relapse	yes

Code	Gender	Age	Status	Th prior the sampling
**CLL01**	M	68	PR	yes
**CLL02**	M	80	new Dg	no
**CLL03**	M	80	new Dg	no
**CLL04**	M	57	relapse	yes
**CLL05**	M	74	progression	yes
**CLL06**	M	76	progression	no
**CLL07**	M	72	progression	yes
**CLL08**	F	70	progression	yes
**CLL09**	F	72	progression	yes
**CLL10**	F	63	progression	yes
**CLL11**	F	74	progression	yes

Supplemental Figure 2: Principal component analysis (PCA) of gene expression data of MCL *vs* healthy controls (a), CLL *vs* healthy controls (b), and MCL *vs* CLL (c). (d) Overview of patient samples included in PCA analysis. PR – partial remission. Note that one MCL patient donated two samples (from peripheral blood and pleural effusion) and these two samples (MCL10a and MCL10b) were closely related on hierarchical clustering (SF1) and PCA (SF2).

Supplemental figures

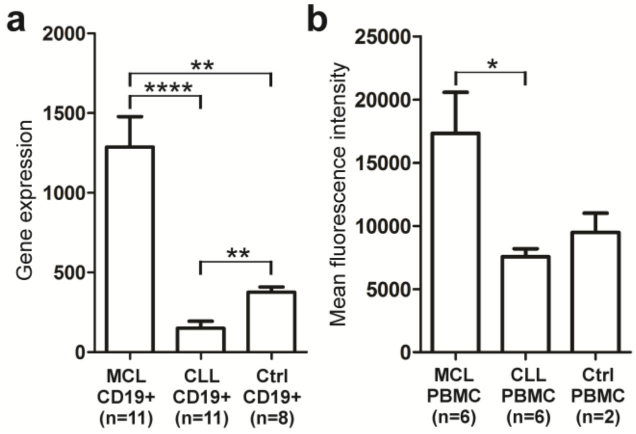

Supplemental Figure 3: Expression of MARCKS in MCL and CLL patients from test group and CD19+ cells from healthy donors determined by microarrays. (a) Expression of MARCKS in MCL and CLL patients from test group and CD19+ cells from healthy donors determined by microarrays. (b) The mean fluorescence intensity of fluorescently labeled anti-MARCKS antibody in PBMCs of MCL and CLL patients from validation group and healthy controls determined by flow cytometry. Data are represented as mean and SEM of fluorescence intensity values. Results of Tukey's HSD statistical test are displayed. *p ≤ 0.05, **p ≤ 0.01, ****p ≤ 0.0001.

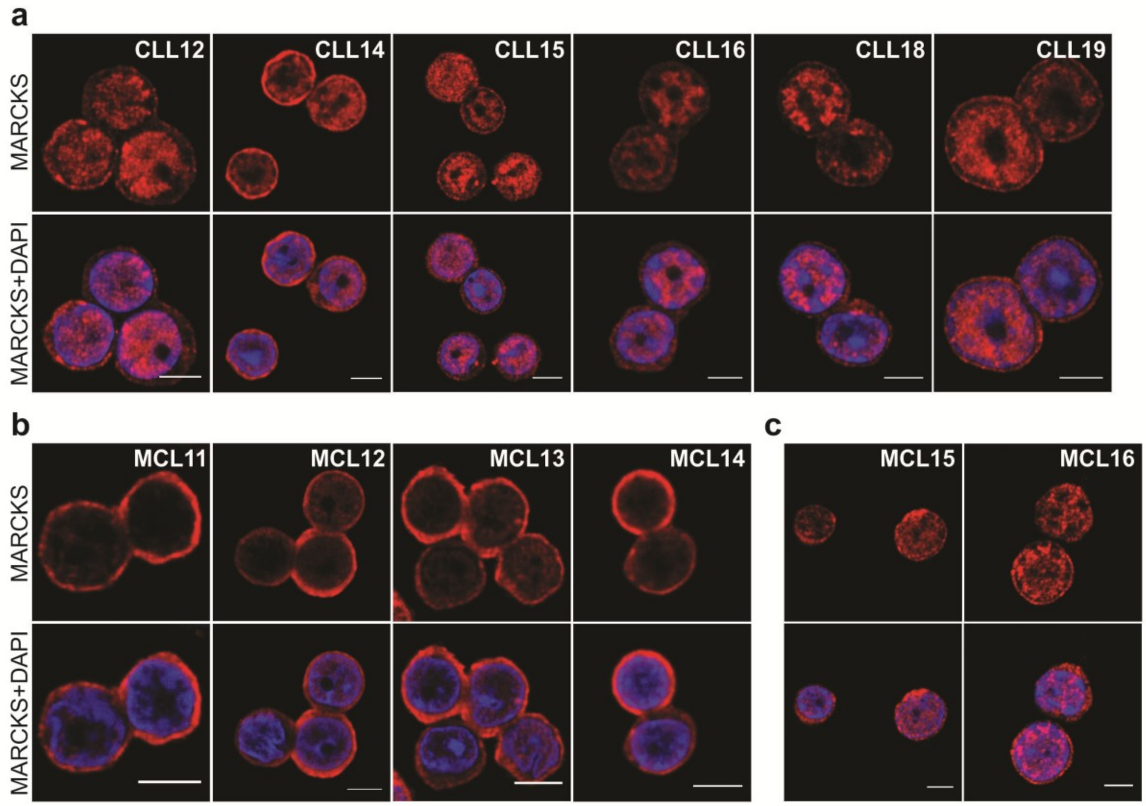

Supplemental Figure 4: MARCKS localization in PBMC of 6 CLL patients (a), 4 MCL patients with peripheral blood leukemization (b), and 2 MCL patients with low abundance of tumor cells in the peripheral blood (c). Cells were fixed and fluorescently labeled for MARCKS. DAPI was used for nuclear staining. Scale bars represent 5µm.

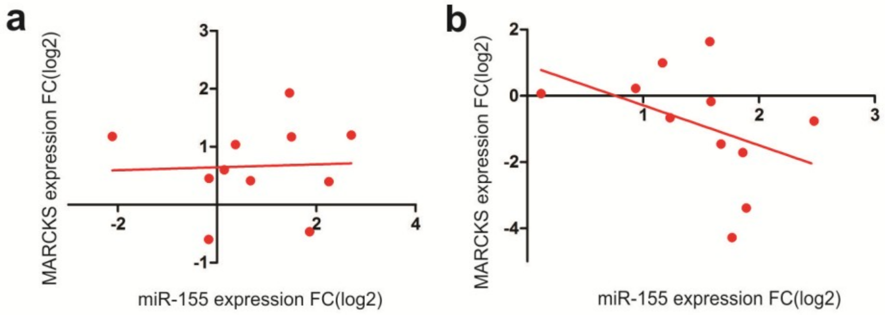

Supplemental Figure 5: Correlation analysis between mRNA expression of MARCKS and miR-155 in (a) MCL (r =0,046, p = 0,894) and (b) CLL (r = -0,418, p = 0,201). (Pearson correlation, two tailed, 95% confidence interval). X and y axes are in log2 scale, a line indicates linear regression curve.

```
   11819                                                              11887
wt TTGCTGTAGGCTGTATGCTGTTAATGCTAATCGTGATAGGGGTTTTTGCCTCCAACTGACTCCTACAT
MM TTGCTGTAGGCTGTATGCTGTTAATG-----------------------CCTCCAACTGACTCCTACAT
BM TTGCTGTAGGCTGTATGCTGTTAATG------------------------------CTGACTCCTACAT
```

Supplemental Figure 6: Sequence of the MIR155HG gene (shown 5`11819 - 11887 3`). Sequence for the mature miR-155 (23 nt) is in bold. Inhibitory sequence for mRNA targets (MiRTarBase) is highlighted by the box. Wt – wild type, MM – monoallelic mutant, BM – biallelic mutant.

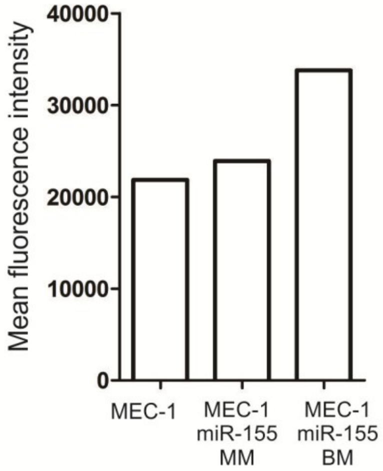

Supplemental Figure 7: The mean fluorescence intensity of anti-MARCKS antibody in MEC-1 cell line and miR-155 mutant MEC-1 clones as determined by flow cytometry.
