# Supplementary material for: Differential expression, localization and activity of MARCKS between mantle cell lymphoma and chronic lymphocytic leukemia
Source: Blood Cancer J. 2016 Sep 23;6(9):e475–. doi: 10.1038/bcj.2016.80 (PMC5056972; doi:10.1038/bcj.2016.80)
Supplement: Supplementary Tables [file bcj201680x3.pdf]

SUPPLEMENTAL TABLES

Supplemental Table 1: Clinical characteristics of test group of patients with MCL.

Patient code	Age/sex	Sample	MIPI	Ann Arbor	Treatment before sampling	PB clonal lymphocytes (%)	Clinical state related to sampling	Cytogenetic aberration additional to t11,14
MCL01	64/F	PB	7	IVB	Yes	NA	PR	NA
MCL02	84/F	PB	8	IVB	No	94	new Dg	none
MCL03	66/M	PB	6.95	IVB	No	NA	new Dg	del11q
MCL04	60/M	PB	7.64	IVB	No	NA	new Dg	del17p
MCL05	57/M	PB	5.54	IVA	No	47	new Dg	none
MCL06	57/M	PB	5.65	IVA	No	NA	new Dg	none
MCL07	76/M	PB	8.5	IVB	No	37	new Dg	NA
MCL08	68/M	PB	8.5	IVB	No	70	new Dg	del17p, del11q
MCL09	41/M	PB	5.38	IVA	No	17	new Dg	none
MCL10a	71/M	PB	10	IVA	Yes	50	relapse	del17p
MCL10b	71/M	PE	10	IVA	Yes	NA	relapse	del17p

PB – peripheral blood, PE – pleural effusion, NA – not assessed, PR – partial remission

Supplemental Table 2: Clinical characteristics of test group of patients with CLL.

Patient code	Age/sex	Sample	RAI	Treatment before sampling	PB clonal lymphocytes (%)	Clinical state related to sampling	Cytogenetic aberration	Mutated genes
CLL01	68/M	PB	2	Yes	NA	PR	del11q	CD38, ZAP-70
CLL02	80/M	PB	3	No	75	new Dg	del13q	ZAP-70
CLL03	80/M	PB	1	No	93	new Dg	del11q, del13q	none
CLL04	57/M	PB	4	Yes	76	relapse	del 17p, del13q	CD38, ZAP-70
CLL05	74/M	PB	2	Yes	100	progression	del 11q, del13q	CD38, ZAP-70
CLL06	76/M	PB	4	No	97	progression	del13q	none
CLL07	72/M	PB	2	Yes	98	progression	del11q, del13q	CD38, ZAP-70
CLL08	70/F	PB	1	Yes	NA	progression	none	IgHV, CD38
CLL09	71/F	PB	2	Yes	97	progression	del 17p, del13q	IgHV, CD38
CLL10	63/F	PB	4	Yes	NA	progression	del11q, del13q	CD38, ZAP-70
CLL11	74/F	PB	3	Yes	100	progression	trisomy 12	ZAP-70

PB – peripheral blood, NA – not assessed, PR – partial remission

Supplemental Table 3: Top 10 identified biological processes in MCL.

GO term	number of DE genes	number of ALL genes	p-value
Immune system process	156	2131	4.80E-15
Cell activation	86	843	4.85E-15
Regulation of immune system process	100	1084	4.85E-15
Leukocyte activation	71	621	8.62E-15
Immune response	107	1294	3.99E-13
Regulation of response to stimulus	181	2936	2.34E-11
Response to stimulus	344	7069	3.23E-11
Lymphocyte activation	58	531	4.51E-11
Response to stress	190	3193	8.38E-11
Defense response	104	1359	9.70E-11

Supplemental Table 4: List of top 10 upregulated mRNAs between MCL and normal B cells.

Gene symbol	Gene name	FC	p-value
CCND1	cyclin D1	155.06	6.44E-11
SOX11	SRY (sex determining region Y)-box 11	127.71	1.05E-08
CNR1	cannabinoid receptor 1 (brain)	27.13	1.70E-05
OTTHUMG00000179333	OTTHUMG00000179333	22.19	3.81E-07
WNT3	wingless-type MMTV integration site family, member 3	16.92	0.001105
ABCA6	ATP-binding cassette, sub-family A (ABC1), member 6	16.17	0.001551
DMD	dystrophin	14	1.91E-06
PNMA2	paraneoplastic antigen MA2	13.25	0.000487
SH3BP4	SH3-domain binding protein 4	12.57	4.51E-06
DBN1	drebrin 1	10.97	0.00011

Supplemental Table 5: List of top 10 downregulated mRNAs between MCL and normal B cells.

Gene symbol	Gene name	FC	p-value
**CD200**	CD200 molecule	0.03	1.38E-09
**VCAN**	versican	0.03	0.00020
**LYZ**	lysozyme (renal amyloidosis)	0.03	0.00285
**SERPINA1**	serpin peptidase inhibitor, clade A (alpha-1 antiproteinase, antitrypsin) 1	0.03	0.00013
**KIAA0125**	KIAA0125	0.04	1.89E-06
**CPVL**	Carboxypeptidase, vitellogenic-like	0.04	1.83E-05
**NT5E**	5'-nucleotidase, ecto (CD73)	0.04	3.55E-06
**ANXA1**	annexin A1	0.04	0.00080
**SESTD1**	SEC14 and spectrin domains 1	0.05	4.57E-06
**CLEC7A**	C-type lectin domain family 7, member A	0.05	5.67E-05

Supplemental Table 6: List of top 10 deregulated pathways in MCL.

Pathway name	p-value
Natural killer cell mediated cytotoxicity	0.001
Osteoclast differentiation	0.004
Amoebiasis	0.004
MicroRNAs in cancer	0.009
Hematopoietic cell lineage	0.011
Transcriptional misregulation in cancer	0.011
Legionellosis	0.011
NF-kappa B signaling pathway	0.013
Leukocyte transendothelial migration	0.016
Regulation of actin cytoskeleton	0.023

Supplemental Table 7: Top 10 identified biological processes in CLL.

GO term	number of DE genes	number of ALL genes	p-value
Regulation of response to stimulus	173	2936	5.33E-05
Immune system process	134	2131	5.33E-05
Actin filament bundle assembly	18	101	1.07E-04
Actin filament bundle organization	18	101	1.07E-04
Leukocyte activation	53	621	1.32E-04
Lymphocyte activation	47	531	2.03E-04
Cell activation	64	843	3.26E-04
Actin filament-based process	47	550	4.25E-04
Intracellular signal transduction	134	2266	4.46E-04
Regulation of hydrolase activity	78	1125	4.57E-04

Supplemental Table 8: List of top 10 deregulated pathways in CLL.

Pathway name	p-value
Chemokine signaling pathway	4.86E-05
MicroRNAs in cancer	3.51E-04
Apoptosis	3.51E-04
Morphine addiction	8.14E-04
Protein processing in endoplasmic reticulum	8.14E-04
Sphingolipid signaling pathway	0.004
Ras signaling pathway	0.004
Cholinergic synapse	0.004
HIF-1 signaling pathway	0.006
Hematopoietic cell lineage	0.006

Supplemental Table 9: Clinical characteristics of validation group of patients with MCL.

Patient code	Age/sex	Sample	MIPI	Ann Arbor	Treatment before sampling	PB clonal lymphocytes (%)	Clinical state related to sampling	Cytogenetic aberration additional to t11,14
MCL13	66/M	PB	6.8	IV A	No	78	New Dg	none
MCL14	71/M	PB	6.82	IV A	No	78	New Dg	none
MCL15	62/M	PB	7.64	IV B	No	97	New Dg	none
MCL16	52/F	PB	7.5	IV B	No	95	New Dg	NA
MCL17	63/M	PB	5.54	III A	No	13	New Dg	IgHV
MCL18	62/M	PB	5.81	IV A	No	13	New Dg	none

PB – peripheral blood, NA – not assessed

Supplemental Table 10: Clinical characteristics of validation group of patients with CLL.

Patient code	Age/sex	Sample	RAI	Treatment before sampling	PB clonal lympho-cytes (%)	Clinical state related to sampling	Cytogenetic aberration	Mutated genes
CLL12	64/M	PB	IV	No	94	New Dg	trisomy12, del13q	IgHV
CLL13	66/M	PB	I	No	90	New Dg	trisomy12	CD38
CLL14	52/M	PB	III	No	96	New Dg	del13q	IgHV
CLL15	76/F	PB	III	No	95	New Dg	NA	NA
CLL16	79/F	PB	IV	No	92	New Dg	trisomy12	CD38
CLL17	67/F	PB	I	No	93	New Dg	del13q, del11q	none
CLL18	47/M	PB	IV	No	89	New Dg	norm	none
CLL19	43/M	PB	IV	No	59	New Dg	trisomy12, del13q, del11q	IgHV

PB – peripheral blood, NA – not assessed

Supplemental Table 11: Differentially expressed genes in pathway "MicroRNA in cancer" in MCL and CLL. The downregulated mRNAs are shown in blue, the upregulated mRNAs are shown in red.

Disease	Differentially expressed genes in pathway "MicroRNA in cancer"
**MCL**	CCND1, STMN1, CDK6, CYP1B1, PTGS2, **MARCKS,** CCND2, DNMT3A, PRKCA, WNT3, BCL2, ST14, RECK, SOX4, MDM4
**CLL**	BMPR2, SOX4, RECK, WNT3, BMF, NOTCH2, RPS6KA5, EZR, BCL2, ST14, FOXP1, BCL2L2, **MARCKS,** PIM1, CDK6, UBE2I, MDM4, CD44, PRKCB, THBS1, PRKCE, FSCN1, FZD3, PIK3CA, IKBKB, VIM, SLC45A3, MCL1, CYP1B1, ATM, CDKN2A, VEGFA, KRAS, ZEB2
